# Efficacy of Transcutaneous Electric Nerve Stimulation on Parotid Saliva Flow Rate in Relation to Age and Gender

**Published:** 2016-09

**Authors:** Manu Dhillon, Srinivasa M Raju, Raviprakash S Mohan, Divya Tomar

**Affiliations:** 1 Dept. of Oral Medicine and Radiology, ITS Centre for Dental Studies and Research, Ghaziabad, Uttar Pradesh, India.; 2 Dept. of Oral Medicine and Radiology, Rama Dental College, Kanpur, Uttar Pradesh, India.; 3 Dept. of Oral Medicine and Radiology, Subharti Dental College, Meerut, Uttar Pradesh, India.; 4 Dept. of Pedodontics and Preventive Dentistry, SBB Dental College, Ghaziabad, Uttar Pradesh, India.

**Keywords:** Electrostimulation, Stimulated Salivary Flow, Transcutaneous Electric Nerve Stimulation (TENS), Age, Gender, Parotid Saliva Flow

## Abstract

**Statement of the Problem:**

Treatment with salivary substitutes and stimulation of salivary flow by either mechanical or pharmacologic methods has side effects and only provides symptomatic relief but no long-lasting results.

**Purpose:**

To assess the effectiveness of extraoral transcutaneous electric nerve stimulation (TENS) as a mean of stimulating salivary function in healthy adult subjects; as well as to determine the gender and age-dependent changes in salivary flow rates of unstimulated and stimulated parotid saliva.

**Materials and Method:**

Hundred patients were divided into two groups; Group I aged 20-40 and Group II aged ≥ 60 years. The TENS electrode pads were externally placed on the skin overlying the parotid glands. Unstimulated and stimulated parotid saliva was collected for 5 minutes each by using standardized collection techniques.

**Results:**

Eighty seven of 100 subjects demonstrated increased salivary flow when stimulated via the TENS unit. Ten experienced no increase and 3 experienced a decrease. The mean unstimulated salivary flow rate was 0.01872 ml/min in Group I and 0.0088 ml/min in Group II. The mean stimulated salivary flow rate was 0.03084 ml/min (SD= 0.01248) in Group I, and 0.01556 ml/min (SD 0.0101) in Group II. After stimulation, the amount of salivary flow increased significantly in both groups (*p*< 0.001). Statistical comparison of the two groups revealed them to be significantly different (*p*< 0.001), with Group I producing more saliva. Gender-wise, no statistically significant difference was seen among the subjects in Group I (*p* = 0.148), and those in Group II (*p*= 0.448). Out of 12 subjects with 0 baseline flows, 7 continued to have no flow. Five subjects observed side effects, although minimal and transient.

**Conclusion:**

The TENS unit was effective in increasing parotid gland salivary flow in healthy subjects. There was age-related but no gender-related variability in parotid salivary flow rate.

## Introduction

Saliva is considered as a crucial fluid for maintenance of oral health and comfort.[1-2] Saliva has antibacterial, lubricant, remineralizing, digestive, soft tissue reparative, buffering, and cleansing properties. Therefore, decreased salivary production or altered salivary composition may result in numerous clinical conditions that affect oral health, comfort, and quality of life.

Xerostomia is a real or perceived decrease in the amount of saliva.[1] Its prevalence in the general population is estimated to range 10-29%, and approximately 40% in adults above the age of 50.[3-4] It occurs more frequently in women than in men.[3] The main three causes of reduced salivary flow are medications, radiation therapy of head and neck cancers, and autoimmune disorders.[1]

Treatment with salivary substitutes and stimulation of salivary flow by either mechanical or pharmacologic methods provide some symptomatic relief but no long-lasting result when active treatment is stopped.[5] Systemic sialogogues, like pilocarpine, work well in some patients; but their unavailability and side effects such as profuse sweating restrict their use. These drugs are also contraindicated in asthma, chronic obstructive pulmonary diseases, cardiac arrhythmias, and patients taking beta blockers.[1] Other treatments of xerostomia include daily gum chewing which leads to increased mastication and electrostimulation by using intraoral devices that yields moderate improvement.[6] High frequency, low intensity ultrasound therapy was also found to be ineffective in stimulating the salivary flow rates.[2]

Recently, acupuncture treatment of patients with xerostomia[4, 7] has been demonstrated to be effective and associated with long-lasting results. But the reluctance of patient to undergo “needle therapy” and unavailability of experienced acupuncturist make this treatment modality difficult. To overcome this shortfall, non-invasive transcutaneous electric nerve stimulation (TENS) device has been used to replace the needles.

As per our knowledge and literature search, only one pilot study has been conducted so far determining the efficacy of TENS in stimulating parotid salivary flow; but there is no study determining its effect in relation to age and gender. Thus, research in this area is minimal and sparse.

The purpose of the present study is to evaluate the effectiveness and safety of recently-developed extraoral TENS on parotid saliva flow rate. The study also determines any gender or age-dependent changes in unstimulated and stimulated parotid salivary flow rate in healthy population. 

## Materials and Method

In this prospective randomized comparative study, the subjects served as their own controls. The study was approved by the ethical clearance board of Kothiwal Dental College and Research Centre, Moradabad, Uttar Pradesh. A total of 100 subjects were randomly allocated into Groups I and II, irrespective of gender. The subjects in Group I were aged 20-40, and in Group II, they were ≥ 60 years. Informed written consent was taken from all patients. 

**Figure 1:**
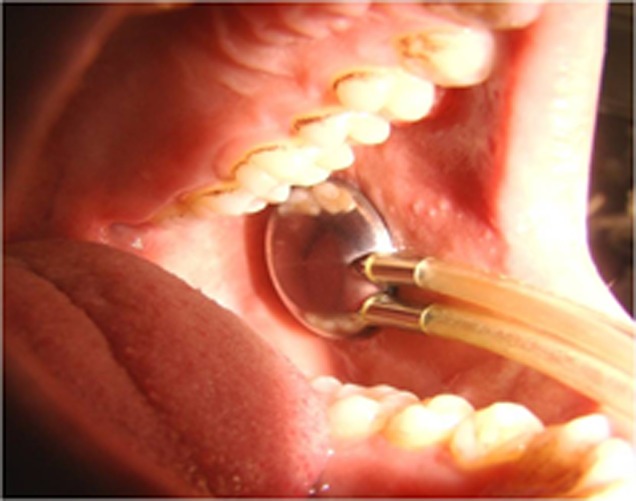
Modified Carlson-Crittenden cup positioned over parotid duct opening.

The exclusion criteria included patients with pacemakers, autoimmune diseases, pregnancy, history of salivary gland pathology, and current use of any medication which noted the incidence of xerostomia on its side-effect profile occurring greater than 1% as listed in the Physician’s Desk Reference. Subjects were refrained from eating, drinking, chewing gums and oral hygiene procedures for at least one hour prior to the appointment. The self-adhesive electrode pads (DURA-STICK II; Chattanooga Group, A Division of Encore Medical, 4717 Adams Road, Hixson, U.S.A.) were placed externally on the skin overlying the parotid glands with the TENS unit (Physio Tens-AT; International Electro Medical Co., New Delhi, India) in the ‘‘off’’ position. The duct orifices were wiped with sterile gauze and located bilaterally with the help of diagnostic instruments. Negative suction was created with the aid of suction bulb attached to the cups. Modified Carlson-Crittenden saliva collection cups (Academic Centre for Dentistry; Van de Boechorststraat 7, 1081BT Amsterdam, The Netherlands) were bilaterally placed over Stensen’s duct (Figure 1).

**Figure 2:**
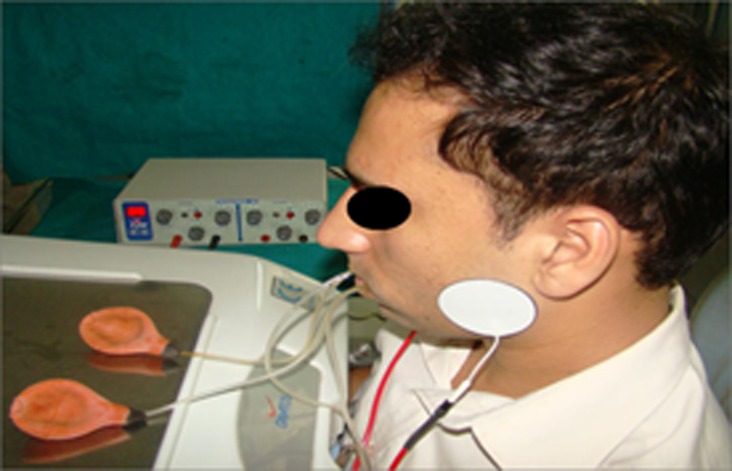
Patient during parotid saliva collection with TENS device on.

The duct orifices on both sides were checked to lie over the inner ring of the cups. Unstimulated saliva was collected into vials for 5 minutes. The cups were then removed from the oral cavity; the remaining saliva was also collected from the tubing in the same vial. The cups were again replaced, the TENS unit was then activated. The pulse rate was fixed at 50 Hz, the pulse duration at 250 µsec, and the unit was in normal mode. The intensity control switch was adjusted for patient comfort. Intensity was turned up 1 increment at a time at 5-second intervals until the subject raised their hand to indicate that an optimal intensity level was reached. Optimal intensity was defined as the maximum intensity that the subject still perceived to be comfortable. Stimulated saliva was then collected into a separate vial for 5 minutes (Figure 2).

All subjects completed the protocol. The statistical analysis was done by using SPSS software (Statistical Package for Social Sciences), version 15.0. The values were represented in number (%) and mean±SD (standard deviation). A log of adverse events was kept. Student’s t-test was used to compare the groups. Correlation analysis was performed to assess the relationship between measurements. For all the tests, *p*≤ 0.05 was considered to be statistically significant.

## Results

The mean age of subjects was 30.84 years in group I and 68.04 years in group II. Eighty seven out of 100 subjects demonstrated increased salivary flow when stimulated via the TENS unit. After stimulation in group I, the salivary flow increased in 43 subjects, decreased in 3 subjects, and remained the same in 4 subjects. In group II, 44 subjects out of 50 demonstrated increased salivary flow after stimulation. In 6 subjects, the salivary flow remained the same; but no subject demonstrated reduced salivary flow after stimulation. In Group I the mean value of salivary flow was 0.0936 ml; while, in Group II the mean value was 0.0440 ml (Table 1).

**Table 1:** Comparison of saliva flow (unstimulated and stimulated) in the two study groups (amount of flow in 5 minutes)

	**Statistic**	**Unstimulated**		**Stimulated**	
		**Group I**	**Group II**	**Group I**	**Group II**
	Mean	0.0936	0.0440	0.1542	0.0778
	SD^#^	0.0411	0.0373	0.0624	0.0505
	Minimum	0	0	0	0
	Maximum	0.22	0.22	0.30	0.24
	"t" ^##^	6.319		6.319	
	"p"^###^	<0.001		<0.001	

#: Standard deviation          ##: t value (Student’s t-test)      ###: p value (Student’s ‘t’ test)

**Figure 3:**
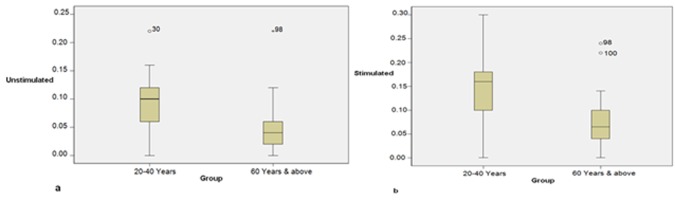
a: Comparison of unstimulated salivary flow in two groups.  b: Comparison of stimulated saliva flow in  study groups.

In both groups, the minimum and maximum amount of flow noted in a patient was, respectively, 0 and 0.22 ml. Irrespective of gender, the mean unstimulated salivary flow was significantly higher in Group I as compared to Group II (Table 1, Figure 3a).

In both groups, the males produced more saliva than females; however gender-wise, no statistically significant difference was seen among the subjects in Group I (*p*= 0.148) and Group 2 (*p*= 0.448) in terms of unstimulated mean salivary flow. Males in group I produced more saliva than males in group II. Similarly females in group I produced more saliva than the females in group II (*p*< 0.001). (Table 2)

**Table 2:** Gender-wise comparison of unstimulated salivary flow (amount of flow in 5 minutes)

**SN**	**Group**	**Males**			**Females**			**"t"**	**"p"**
		**N**	**Mean**	**SD**	**N**	**Mean**	**SD**		
1.	I	41	0.0976	0.0410	9	0.0756	0.0384	1.472	0.148
2.	II	39	0.0462	0.0409	11	0.0364	0.0196	0.765	0.448
Group I vs. II									
1.	"t"	5.611			2.955				
2.	"p"	<0.001			0.008				

In both groups, the minimum amount of stimulated salivary flow noted in a patient was 0 ml; whereas, the maximum amount of flow noted in Group I was 0.30, and in Group II it was 0.24 ml. Irrespective of gender, the mean stimulated salivary flow was found to be significantly higher in Group I as compared to Group II (*p*< 0.001). (Figure 3b)

Both males and females in Group I had significantly higher mean salivary flow as compared to those in Group II. (Table 3) In both groups, the amount of salivary flow increased significantly after stimulation (*p*< 0.001). (Table 4) The mean unstimulated salivary flow rate was 0.01872 ml/min in Group I, and 0.0088 ml/min in Group II. The mean stimulated salivary flow rate in Group I was 0.03084 ml/min, and in Group 2 it was 0.01556 ml/min. (Table 5)

**Table 3:** Gender-wise comparison of stimulated salivary flow (amount of flow in 5 minutes)

**SN**	**Group**	**Males**			**Females**			**"t"**	**"p"**	
		**N**	**Mean**	**SD**	**N**	**Mean**	**SD**			
1.	I	41	0.1598	0.0609	9	0.1289	0.0664	1.355	0.182	
2.	II	39	0.0815	0.0536	11	0.0645	0.0362	0.986	0.329	
Group I vs. II										
1.	"t"	6.083			2.762					
2.	"p"	<0.001			0.013					

**Table 4:** Change in salivary flow after stimulation (amount of flow in 5 minutes)

**SN**	**Group**	**Before Stimulation**			**After Stimulation**			**"t"**	**"p"**
		**N**	**Mean**	**SD**	**N**	**Mean**	**SD**		
1.	I	50	0.0936	0.0411	50	0.1542	0.0624	10.600	<0.001
2.	II	50	0.0440	0.0373	50	0.0778	0.0505	11.037	<0.001

**Table 5:** Salivary flow per minute

**SN**	**Group**	**Before Stimulation**			**After Stimulation**			**"t"**	**"p"**
		**N**	**Mean**	**SD**	**N**	**Mean**	**SD**		
1.	I	50	0.01872	0.00822	50	0.03084	0.01248	10.600	<0.001
2.	II	50	0.0088	0.00746	50	0.01556	0.0101	11.037	<0.001

There was no adverse event or long-term side effect to the use of TENS. Five subjects (5%) experienced side effects. The side effects included numbness or anesthesia of skin adjacent to the electrodes (n=3), twitching of the facial musculature (n=1), and itching over the skin where the electrodes were applied (n=1). These effects were transient and ceased immediately once the TENS unit was turned off. 

## Discussion

Electrostimulation to produce saliva was studied in the past and showed moderate promise, but never became part of the mainstream therapy. Research in this area has been sparse as a result. Several studies were conducted to see the efficacy of electrostimulation in increasing the salivary flow. Yet, only one pilot study has been conducted so far to demonstrate the TENS unit as a means of stimulating salivary production. In this study, an extraoral device has been studied as a means of stimulating parotid salivary flow through TENS device. The study also compared the effect of extraoral TENS in two age groups (20-40, and ≥ 60 years), simultaneously evaluating the gender differences.

There was great variability in the amount of saliva produced in our study. Some of the subjects demonstrated no flow initially. This was not surprising, as 21-22% of the population demonstrate no parotid flow even when measured over 5 minutes_­_.[8] The wide variation of salivary flow rates in this study was within normal limits as reported in the literature.[6, 8]

The subjects that seemed to demonstrate significant change were those with initial saliva flow already present. In 7 out of 12 cases, the TENS was unable to stimulate saliva where the salivary flow was 0 at baseline. Since, it is the serous component of parotid saliva that confers the greatest protection against dry mouth; one may claim that TENS would not be useful. These findings also suggest that TENS, by itself, is less likely to be effective in cases where there is no baseline saliva flow such as in long-standing Sjögren’s syndrome or high-dose radiation therapy where complete destruction of the salivary gland unit has occurred. This is a shortcoming that would concern other current treatment modalities.

Meanwhile, in cases with residual salivary function, the TENS appears to be effective. These findings are suggestive that TENS may work quite well or even synergistically with other sialogogues. TENS may act more efficiently as an accelerator of salivary flow rather than an initiator. Therefore, it is likely to be more effective in cases of decreased salivary gland function rather than absolute absence of function.

In 3 subjects, salivary flow decreased with TENS, and in 3 others, it remained the same. The mechanism for this may involve the frequency and intensity settings and whether the brain perceived the stimulus as being painful. Typically, the salivary reflex is enhanced when nociceptive input reaches the brain via the trigeminal sensory nuclei.[9] However, not all preganglionic parasympathetic fibers are necessarily facilitated, some may be inhibited. This study did not evaluate the intensity and frequency that produces the maximum volume of saliva. We attempted to minimize these effects by keeping the stimulus at a tolerable level.

The effectiveness of TENS in stimulating salivary flow depended on age. Although, the literature has shown that salivary flow does not diminish with age,[6] our results are not in agreement with this observation. There was no statistically significant difference between genders as reported in previous studies.[10] Preceding investigations do not support statistically significant gender differences in terms of salivary output even though females have a tendency to produce less saliva.[6] This study had a limited number of female participants. Based on the lack of gender differences noted in the literature, we did not actively involve females in the study. Nonetheless, based on our findings, future studies can be conducted involving more female participants.

Some of the side effects of TENS therapy, noted in this study, included twitching of the facial musculature, itching over the site of electrode application, and anesthesia of the facial skin; although they were minimal and transient. Some of them could be minimized by adjusting the electrode location. These effects ceased once the TENS unit was turned off and the electrodes were removed. Perhaps modifications (such as using smaller electrodes) can be made to future TENS units, to minimize the side effects and make electrostimulation of the parotids more effective. TENS has a long proven safety record and has been used in some cases of pediatric dental anesthesia[11] and physical therapy centers.

Weiss *et al.* in 1986,[12] and Steller *et al.* in 1988[13] used electrostimulation device in groups of dry mouth sufferers, and Talal *et al.* in 1992[14] used TENS acupuncture in patients with radiation-induced xerostomia. All the studies suggested that electrostimulation increases salivary flow. However, the employed saliva collection methods are subjective and through expectoration which are not completely reliable. The collection method used in our study was through modified Carlson-Crittenden cups[15] which are more reliable and accurate. Subjective measures of the amount of saliva were not recorded. Owing to the collection method, the mouth remained passively open during the study, which may have produced drying effects that would have influenced subjective measurements.

The mechanism by which the TENS unit worked on the parotid gland is not clear. It is possible that it directly stimulated the auriculotemporal nerve that supplies secretomotor drive to the parotid gland. It is unclear if peripheral stimulation of the gland results in a reflex facilitation of central output from the salivatory nucleus of the medulla. The early investigators of electrostimulation postulated that normal physiologic salivary reflexes are augmented.[16] Salivation is controlled by both sympathetic and parasympathetic efferent nerves.[17] Sympathetic stimulation produces sparse, viscous saliva. In order to electrically stimulate sympathetic salivation, higher frequencies and longer pulse duration is required.[18]On the other hand, electric stimulation of parasympathetic nerves of the salivary glands produces copious amounts of watery saliva at lower frequencies, and it is this voluminous, serous saliva of the parotid gland that would be clinically most useful for managing xerostomia.[16] Within this dual autonomic system it is clear that salivation is primarily under parasympathetic control.

One advantage of this technique over previous modalities of electrostimulation is that the TENS unit is an extraoral device. Thus, the potential for salivary production while eating would be beneficial. That was not possible with the intraoral devices. Another advantage is that previous electrostimulators were expensive but TENS unit in this study was affordable. Portable TENS devices are also currently easily available. Fox *et al.* in 1991[19] and Vivino *et al.* in 1999[20] concluded that cholinergic agonist pilocarpine work well for some patients but often have undesirable side effects like profuse sweating. On the other hand, there are no such side effects in TENS therapy. However, neither TENS nor pilocarpine can increase the function of glands that are completely damaged by irradiation.[21] Dodds *et al.* in 1991[6] and Jenkins and Edgar in 1989[22] indicated that daily gum-chewing over a prolonged time period resulted in functional increase in parotid salivary flow. The main advantage offered by TENS over other nonpharmacological measures such as chewing gum or citric lozenges is that it may be used while eating. Chewing gum have shown mixed results in previous studies,[22] but needs to be avoided in those with temporomandibular disorders. Artificial saliva preparations are often objectionable.

One important shortcoming of the study is the placement of electrodes which was done approximately over the parotid region without exact anatomic measurements. But the diameter of the electrodes used in the study was large enough (6 cm) to overcome this limitation, as compared to the size of parotid gland. 

To our knowledge, this is one of the few studies demonstrating the potential of TENS for increasing the salivary flow. Furthermore, our saliva collection method was more reliable than those used in previous electrostimulation studies, which were very subjective and prone to contamination by nasal and gastric secretions as well as food debris. 

## Conclusion

The results presented in normal healthy subjects warrant further study of TENS therapy in a cohort of dry mouth sufferers. Aspects for future study should include the duration of the increase in salivary flow rate after cessation of TENS, the ability of TENS to stimulate parotid salivary flow specifically when there is none at the baseline, patient’s acceptance, subjective measures, and usefulness of TENS alone versus in combination with other sialogogues. This technique may not work in every individual, but individuals with intense symptoms of dry mouth may benefit from it. TENS may be an additional modality in an ever-growing armamentarium to manage salivary gland dysfunction. 
